# X-ray photon correlation spectroscopy using a fast pixel array detector with a grid mask resolution enhancer

**DOI:** 10.1107/S0909049512038769

**Published:** 2012-09-28

**Authors:** Taiki Hoshino, Moriya Kikuchi, Daiki Murakami, Yoshiko Harada, Koji Mitamura, Kiminori Ito, Yoshihito Tanaka, Sono Sasaki, Masaki Takata, Hiroshi Jinnai, Atsushi Takahara

**Affiliations:** a ERATO, Takahara Soft Interfaces Project, Japan Science and Technology Agency, CE80, Kyushu University, 744 Motooka, Nishi-ku, Fukuoka 819-0395, Japan; b RIKEN/SPring-8 Center, 1-1-1 Kouto, Sayo, Hyogo 679-5148, Japan; c Graduate School of Science and Technology, Kyoto Institute of Technology, Matsugasaki Hashiue-cho 1, Sakyo-ku, Kyoto 805-8585, Japan; d Institute for Materials Chemistry and Engineering, Kyushu University, 744 Motooka, Nishi-ku 819-0395, Japan

**Keywords:** X-ray photon correlation spectroscopy, grid mask resolution enhancer

## Abstract

The performance of a fast pixel array detector with a grid mask resolution enhancer has been demonstrated for X-ray photon correlation spectroscopy (XPCS) measurements to investigate fast dynamics on a microscopic scale. A detecting system, in which each pixel of a single-photon-counting pixel array detector, PILATUS, is covered by grid mask apertures, was constructed for XPCS measurements of silica nanoparticles in polymer melts. The experimental results are confirmed to be consistent by comparison with other independent experiments. By applying this method, XPCS measurements can be carried out by customizing the hole size of the grid mask to suit the experimental conditions, such as beam size, detector size and sample-to-detector distance.

## Abstract 2

The performance of a fast pixel array detector with a grid mask resolution enhancer has been demonstrated for X-ray photon correlation spectroscopy experiments.

## 1. Introduction

X-ray photon correlation spectroscopy (XPCS) uses partially coherent X-rays to provide experimental access to a variety of microscopic dynamic phenomena (Grübel *et al.*, 2008 ▶). If a random arrangement of scatterers is illuminated by coherent radiation, the scattered intensity exhibits a so-called speckle pattern that reflects the instantaneous configuration of the scatterers. Movement of the scatterers causes a corresponding change in the speckle pattern, which thus contains information about the dynamics of the system. To detect intensity fluctuations in the speckle pattern, an XPCS detector must have a spatial resolution comparable with the speckle size and a time resolution that is more rapid than the scatterers’ motion. Although a time resolution of 50 ns has been achieved to date by point detectors (Sikharulidze *et al.*, 2002 ▶), the long X-ray illumination during measurement at various scattering vectors *q* often damages the sample. In contrast, two-dimensional (2D) detectors, which enable simultaneous measurement at various *q* values, reduce the exposure time and thus can prevent such X-ray damage. However, the performance requirements for 2D detectors for XPCS experiments are stringent: high spatial resolution, high speed and high sensitivity. Furthermore, it is desirable that a 2D detector cover a wide *q* region. Specifically in our case, the main target of detection is relatively fast (∼ms) and microscopic (less than 10 nm), such as the local dynamics of polymer chains.

Up to now, considerable numbers of experiments have been conducted with various types of 2D detectors, such as the directly illuminated X-ray CCD (Lumma *et al.*, 2000 ▶), the indirectly illuminated X-ray CCD (Shinohara *et al.*, 2010 ▶) and photon-counting 2D detectors such as the Medipix2 detector (Caronna *et al.*, 2008 ▶), the PILATUS detector (Westermeier *et al.*, 2009 ▶) and the MAXIPIX detector (Orsi *et al.*, 2012 ▶). Among them photon-counting 2D detectors have suitable features for our purpose of measuring the fast dynamics in the microscopic region, *i.e.* a short readout time free from readout noise and wide dynamic range. The pixel size of photon-counting 2D detectors is 55 µm × 55 µm in Medipix and MAXIPIX, and 172 µm × 172 µm in PILATUS, and XPCS experiments have been successfully performed by changing the sample-to-detector distance taking into consideration speckle sizes. On the other hand, for measurements up to the high-*q* region, shortening the sample-to-detector distance is one of the most effective solutions. However, this modification is expected to make XPCS measurements difficult because the speckle size decreases with decreasing distance; thus, a higher spatial resolution is required in 2D detectors. To overcome this problem a method using a grid mask with small holes, which enables measurement up to high-*q* regions, is proposed in this work, and XPCS measurements are demonstrated. The advantage of this method is ‘flexibility or adjustability’ for time- and *q*-ranges in users’ target phenomena on demand. It is to be noted that the method is also widely applicable for detectors such as MAXIPIX and CCDs, not only for PILATUS. Although the developments of faster 2D detectors with smaller pixel size than mentioned above will be continued, the mask method will also be applicable for such detectors and especially meet the urgent demand of only enhancing spatial resolution. To our knowledge this is the first demonstration of the XPCS measurement using a grid mask resolution enhancer although a method of aperturing pixels for the AGIPD (adaptive gain integrating pixel detector) has been proposed by Potdevin & Graafsma (2011 ▶).

In the demonstration we used a PILATUS 100K detector (DECTRIS). The PILATUS detector is a 2D hybrid pixel array detector (Schlepütz *et al.*, 2005 ▶; Broennimann *et al.*, 2006 ▶; Kraft *et al.*, 2009 ▶) that can be operated with a frame rate of up to 300 Hz with no readout noise for single-photon counting; these features are excellently suited to XPCS measurements. Westermeier *et al.* (2009 ▶) successfully conducted XPCS measurements by using quite a long sample-to-detector distance (7.050 m) so that speckles become large enough, comparable with the PILATUS pixel size of 172 µm × 172 µm, to detect at the detector position using the PILATUS 2M detector, which consists of 3 × 8 single modules. Although their solution worked well, such a long sample-to-detector distance and a correspondingly large PILATUS detector, which should increase in size with increasing sample-to-detector distance, may not always be available. In contrast, our method, which uses a grid mask resolution enhancer, can be applied more compactly.

This paper is organized as follows. The details of our XPCS experiments and samples are explained in §2. In §3.1 the validity of our XPCS system is confirmed in the small-*q* region, which can be measured by dynamic light scattering (DLS) with visible light. Measurements in the higher-*q* region, which can be reached only by using X-rays, are described in §3.2. Our conclusions are presented in §4.

## 2. Experimental

The grid mask resolution enhancer demonstrated here should be designed to fit the parameters of the XPCS experimental set-up. The design procedure and experimental set-up are described below.

**Figure 1:**
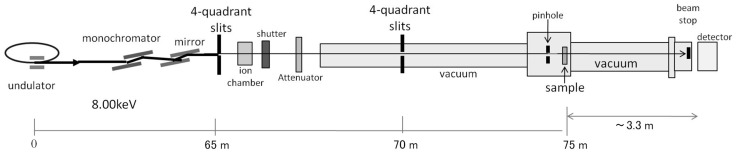
Schematic drawing of the experimental set-up for XPCS at undulator beamline BL19LXU at SPring-8.

XPCS measurements were conducted at the beamline BL19LXU with a 27 m-long undulator at SPring-8 (Japan). Details of the beamline optics are presented elsewhere (Yabashi *et al.*, 2001 ▶). Although the main part of the set-up is almost the same as our previous study (Hoshino *et al.*, 2011 ▶), the set-up is described again here because some parts were changed for this study. Fig. 1 ▶ shows a schematic of our XPCS set-up. The undulator source and monochromator were tuned so that the incident X-ray beam had a photon energy of 8.00 keV (λ = 1.55 Å). The higher harmonic X-rays were removed by the Pt-coated mirrors. The energy bandwidth of the Si(111) monochromator yielded a longitudinal coherence length, ξ_l_ = λ^2^/Δλ ≃ 1 µm. The beam size of the light source was σ ≃ 113 µm × 14 µm (H × V), and the distance between the light source and the sample was *R* ≃ 75 m. From ξ_t_ = λ*R*/(2πσ) the transverse coherence length obtainable at the sample was ξ_t_ ≃ 16 µm × 132 µm (H × V). To make sure of transverse coherence, the beam size was reduced to 20 µm × 20 µm at approximately 10 m upstream of the sample. If the slits position is regarded as an imaginary light source, the vertical transverse coherence could be estimated as ξ_t_ ≃ 78 µm from the equation ξ_t_ = λ*R*/σ for a uniform rectangular source (van der Veen & Pfeiffer, 2004 ▶). Parasitic scattering was shielded by the four-quadrant slits and the pinhole (200 µm in diameter), and spatially coherent X-rays illuminated the sample in a vacuum. The typical flux at the sample was ∼10^10^ photons s^−1^.

The scattered X-rays were detected by a PILATUS 100K detector mounted at ∼3.3 m downstream of the sample. The speckle size is given by σ = (λ*d*)/*a*, where λ is the wavelength, *d* is the sample-to-detector distance, and *a* is the width of the illuminated area. In this set-up the speckle size was estimated to be about 13 µm with a beam size of about 40 µm (FWHM) on the sample, which was estimated by a beam monitor (Hamamatsu Photonics). The pixel size of the PILATUS, 172 µm × 172 µm, was too large to detect the intensity fluctuations of the speckles. To detect these fluctuations the illuminated area of each pixel was narrowed to about 45 µm in diameter by the holes bored in the tantalum foil (NTT-AT) placed in front of the illuminated pixels. These holes ensured the speckle contrast, *A*, which is larger than 0.01, as described in §3.1, inferring that the tolerant data can conventionally be obtained when *A* > 0.01 (Sutton, 2008 ▶). The signal-to-noise ratio (SNR) is proportional to *A* and the number of counts according to Falus *et al.* (2004 ▶). While the SNR for the 45 µm hole was one-fifth of the value estimated for ‘hole size ≃ speckle size’ as a result of the reduced contrast (1/45) and the increase in number of counts (nine times), a hole size of 45 µm was adopted to achieve the aim of carrying out XPCS for fast dynamics at high *q* without the average number of pulses detected in a sampling time being much below unity. The detailed effect of the large hole size adopted here will be discussed in §3.2. The grid mask is 50 µm thick in order to reduce the number of photon counts to less than unity at the covered area. The thickness was estimated from absorbance of tantalum for the maximum number of photon counts controlled to 10^6^, which can be detected with PILATUS for one pixel in a frame.

**Figure 2:**
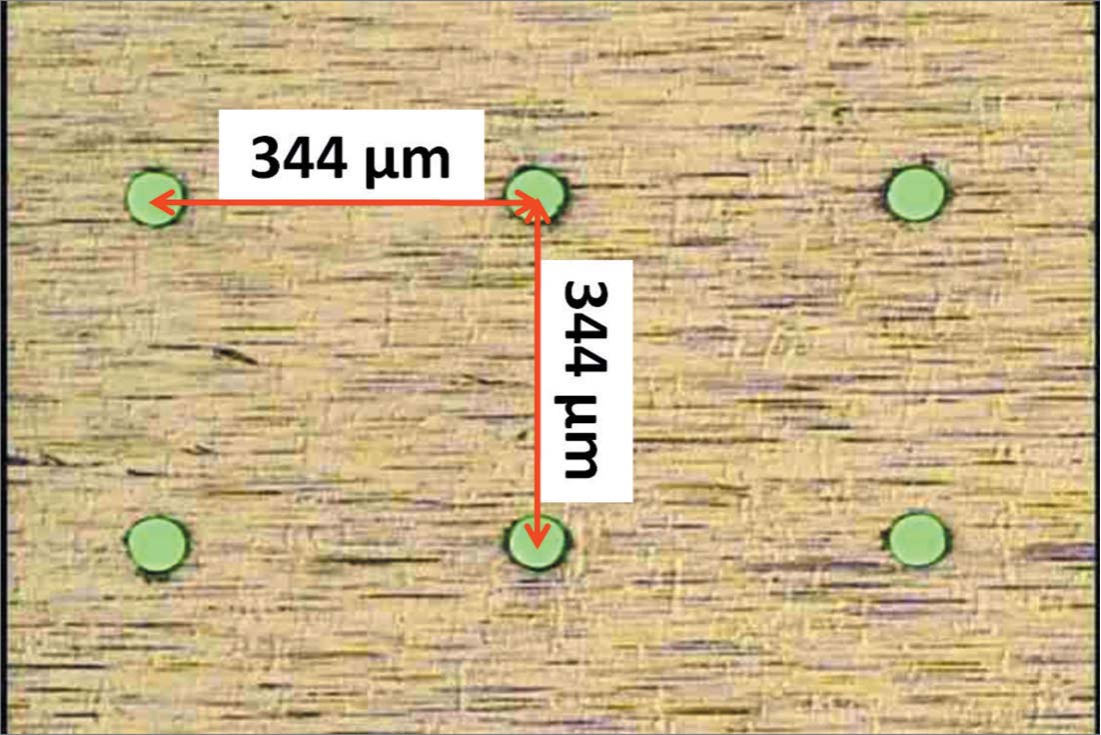
Optical microscope image of the grid mask. The holes (diameter ∼45 µm) are bored at an interval of 344 µm.

**Figure 3:**
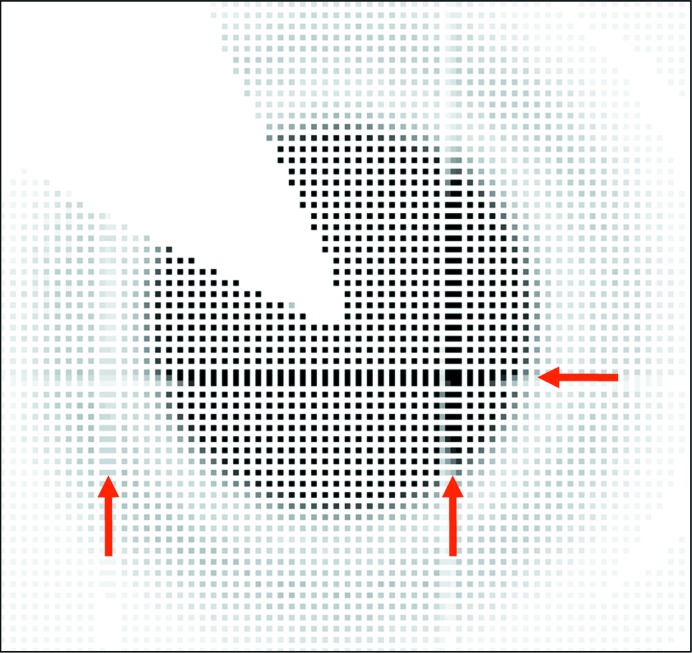
Two-dimensional image for silica particles around the beam center taken by the PILATUS detector after adjustment of the position of the grid mask. Illuminated pixels at intervals of one pixel ensure the hole positions of the grid mask to be exactly adjusted to each pixel. The continuous detected areas, shown by the arrows, correspond to the borders of the CMOS readout chips.

As shown in Fig. 2 ▶, the holes were bored at 344 µm intervals, which is twice the distance between the centers of the pixels, 172 µm. Making intervals enables the positions of the holes to be accurately adjusted for each pixel by checking the measured images, although it reduces the SNR by the reduction of the number of pixels. The adjustment of the position of the grid mask was carried out by checking the scattering images from silica particles, and the 2D image around the beam center after the adjustment is shown in Fig. 3 ▶. Illuminated pixels at intervals of one pixel are shown, which confirm that the positions of the holes are accurately adjusted for each pixel. The continuous detected areas, indicated by arrows in Fig. 3 ▶, correspond to the borders of the complementary metal-oxide semiconductor (CMOS) readout chips (the PILATUS 100K has arrays of 8 × 2 chips) (Kraft *et al.*, 2009 ▶). These areas are excluded from the following analyses.

In XPCS measurements the path difference of the scattered X-rays should be smaller than ξ_l_, which is expressed by the term ξ_l_ > (λ/2π)*qs* and ξ_l_ > 2δ(*q*λ/4π)^2^, where *q* is the scattering vector, *s* is the beam size and δ is the sample thickness. If *s* = 40 µm and δ = 2 mm, the measurable *q*-ranges are expressed as *q* < 1.01 nm^−1^ and *q* < 1.28 nm^−1^, respectively. In our set-up the covered *q*-range by the detector is 4.0 × 10^−3^ < *q* < 4.0 × 10^−1^ nm^−1^, which satisfies the required conditions estimated from ξ_l_. The measurable maximal *q* value of this XPCS system is more than ten times larger than that used for the conventional DLS measurements.

The validity of our XPCS system was confirmed using a dilute suspension of silica particles (200 nm in diameter; Nissan Chemicals) suspended in polypropylene glycol with an average molecular weight of 4000 g mol^−1^ (PPG4000; Wako Chemical) at a volume fraction of 0.5 vol%. The suspension was sealed in a thin-wall glass capillary tube (2 mm in diameter). The sample thickness, *l*, was determined from the absorption of PPG4000 so that the sample transmission is close to the optimum value of ∼1/*e*, calculated from exp(−μ*l*/ρ) where the quotient μ/ρ is called the mass absorption coefficient. Ten thousand images were taken at exposure times of 30 ms, followed by readout times of 3 ms. The same suspension was also examined by DLS with an ALV gonio­meter using an ALV 5000 correlator system (ALV Langen, Germany) employing a HeNe laser (wavelength λ = 632.8 nm, 22 mW). In both experiments the samples were kept at a temperature *T* = 313.15 K.

In order to discuss the experimental data at higher *q*, we also conducted an experiment with a concentrated particle system, which was expected to produce a strong scattering intensity in this *q* range: silica particles (110 nm in diameter) grafted with a PS brush (*M*_n_ = 5.53 × 10^4^ g mol^−1^, *M*_w_/*M*_n_ = 1.78, graft density = 0.48 chains nm^−2^) with a silica concentration of 22.4 vol%. The sample was prepared following the method of Matsuda *et al.* (2008 ▶). In the XPCS measurements the sample was kept in a capillary tube with a diameter of 1 mm at 473.15 K (the sample thickness was thinner than the optimum value to avoid the thermal gradient), and 20000 images were taken with an exposure time of 30 ms, followed by a readout time of 3 ms. The static properties of this sample were investigated by small-angle X-ray scattering conducted at beamline BL19B2 at SPring-8 at an incident X-ray wavelength λ of 0.0689 nm with a sample-to-detector distance of 41890 mm.

## 3. Results and discussion

### 3.1. Evaluation of the measured time-autocorrelation function

In this subsection the validity of our XPCS system is confirmed using the dilute particle suspension.

The scattering intensity *I*(**q**, *t*) at a scattering vector **q** and time *t* was obtained from the counts in each pixel of the temporal images of silica particles in PPG4000. The intensity time-autocorrelation function *g*_2_(**q**, *t*) at each pixel was then evaluated as
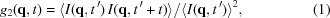
where the angular brackets indicate time-averaging. In the evaluation, *g*_2_(**q**, *t*) is azimuthally averaged over all pixels within rings belonging to |δ**q**| = 0.41 × 10^−2^ nm^−1^ as
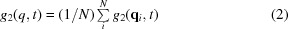
with
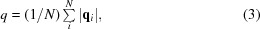
where **q**_*i*_ is the wavevector at the *i*th pixel and *N* is the number of pixels in an averaged ring. In conventional XPCS measurements with 2D detectors (Lumma *et al.*, 2000 ▶) the correlation function is normalized by the azimuthally averaged intensity to investigate slower dynamics in comparison with the measurement time such as aging dynamics (Madsen *et al.*, 2010 ▶). However, the same procedure was not applied in the present system, where the hole sizes of the grid mask are not strictly uniform and vary with a standard deviation of 3.69 µm, and consequently the baseline obtained by considering the limit 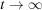 is always larger than unity without physical meaning. Therefore we normalized at each pixel as written in (1), which does not require a strictly uniform hole size. In that case the baseline of *g*_2_(*q*, *t*) is always close to 1 as 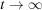, following the primitive definition of the autocorrelation function.

**Figure 4:**
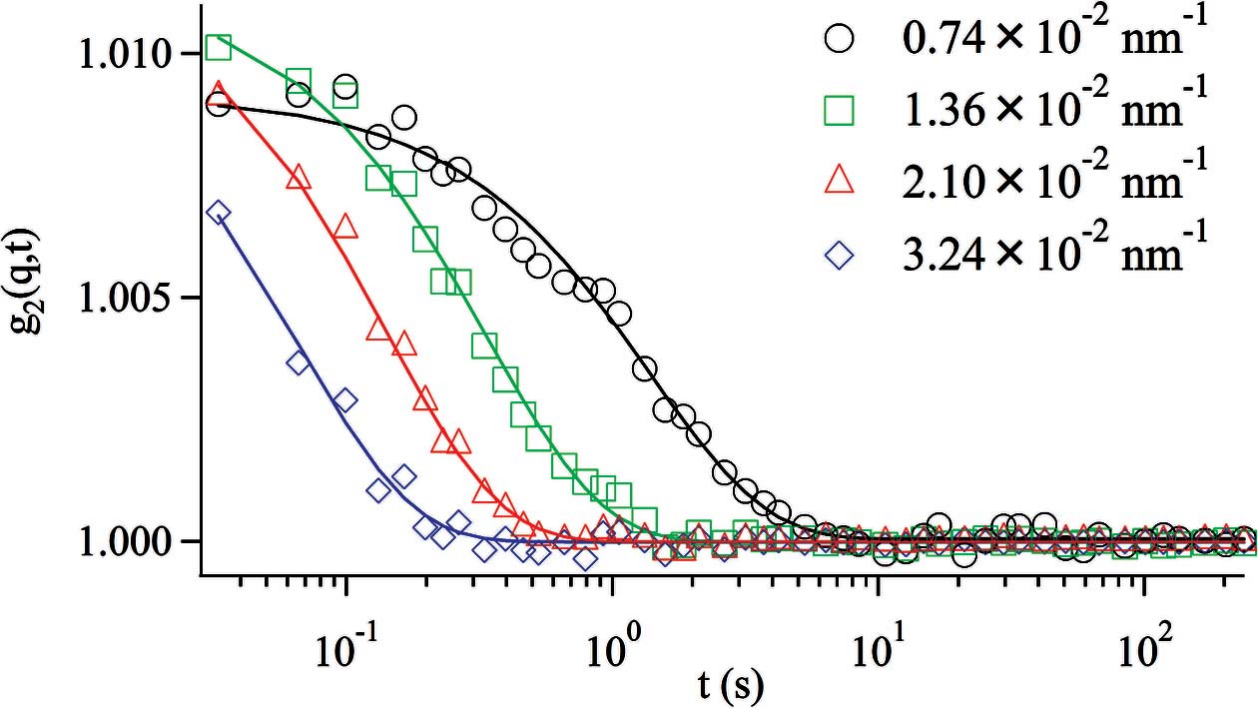
Normalized intensity time-autocorrelation function obtained from scattering of silica particles in PPG4000 at 313.15 K. The solid lines are fitting curves by equation (4).

**Figure 5:**
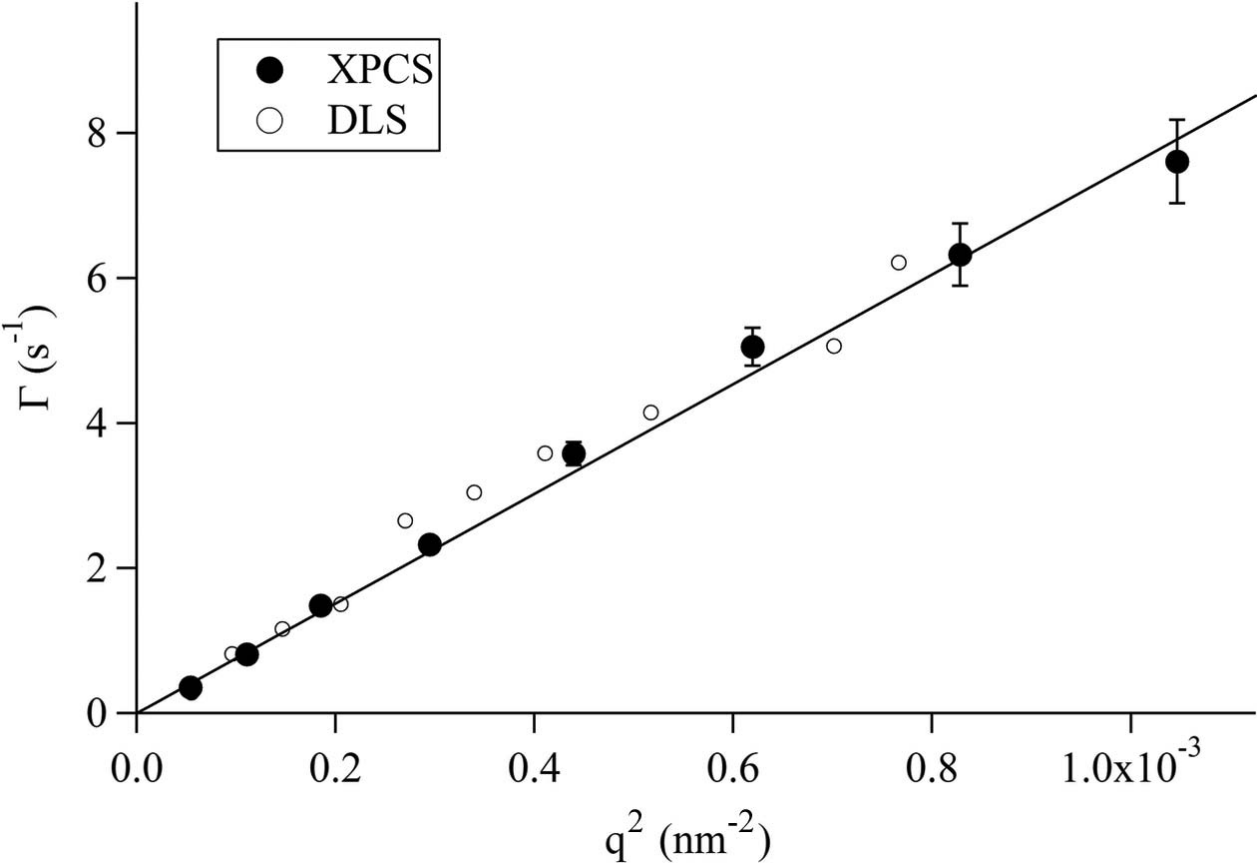
Relaxation rates Γ measured by XPCS (filled circles) and DLS (open circles) *versus* *q* ^2^ for silica particles in PPG4000 at 313.15 K. The solid line represents the fitting curve for XPCS data by a proportional equation.

Fig. 4 ▶ shows typical examples of the measured *g*_2_(*q*, *t*) for silica particles in PPG4000. For a Brownian diffusion process the expected functional form of the correlation function is (Berne & Pecora, 1975 ▶)
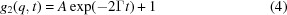
with
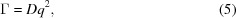
where *D* is the diffusion coefficient. All the data measured by XPCS are fitted well by (4), as shown by the solid lines in Fig. 4 ▶, from which Γ = 0.36, 1.48, 5.05 and 7.39 s^−1^ is obtained at *q* = 0.74 × 10^−2^, 1.36 × 10^−2^, 2.10 × 10^−2^ and 3.24 × 10^−2^ nm^−1^, respectively. The measured Γ values are plotted as a function of *q* ^2^ in Fig. 5 ▶, and the linear relationship between the measured Γ and *q* ^2^ shows excellent agreement with that predicted for Brownian motion [*i.e.* equation (5) above].

The dynamical behavior of the same sample was also measured by DLS (open circles in Fig. 5 ▶). The Γ measured by XPCS should coincide with that measured by DLS unless multiple scattering occurs in the DLS measurement. The *g*_2_(*q*, *t*) measured by DLS are also well fitted by equation (4). The Γ values obtained from the DLS measurements, plotted in Fig. 5 ▶, show excellent agreement with those measured by XPCS, clearly demonstrating the validity of the measured data from the present XPCS system. In addition, we note that the suspension was proven to be dilute enough not to cause multiple scattering in DLS.

The prefactor *A* obtained in the XPCS experiment is about 0.01, which denotes the speckle contrast. The speckle contrast can be approximated by *A* = 1/[1 + (*P*/σ^2^)^2^], where *P* is the effective pixel area and σ^2^ is the speckle area. In the present study *P* is the area of a circle with a diameter of 45 µm, and σ was estimated in the experimental section. Using these values the theoretically predicted *A* is estimated to be about 0.011, which is in good agreement with the measured value. The speckle contrast can be enhanced by reducing the beam size when the number of detected counts is high enough and more high contrast is desired.

### 3.2. Demonstration of measurements in the X-ray region

The concentrated particle system consisting of PS grafted particles was used to examine the performance of our XPCS system at a higher *q* region than that reachable by visible light.

**Figure 6:**
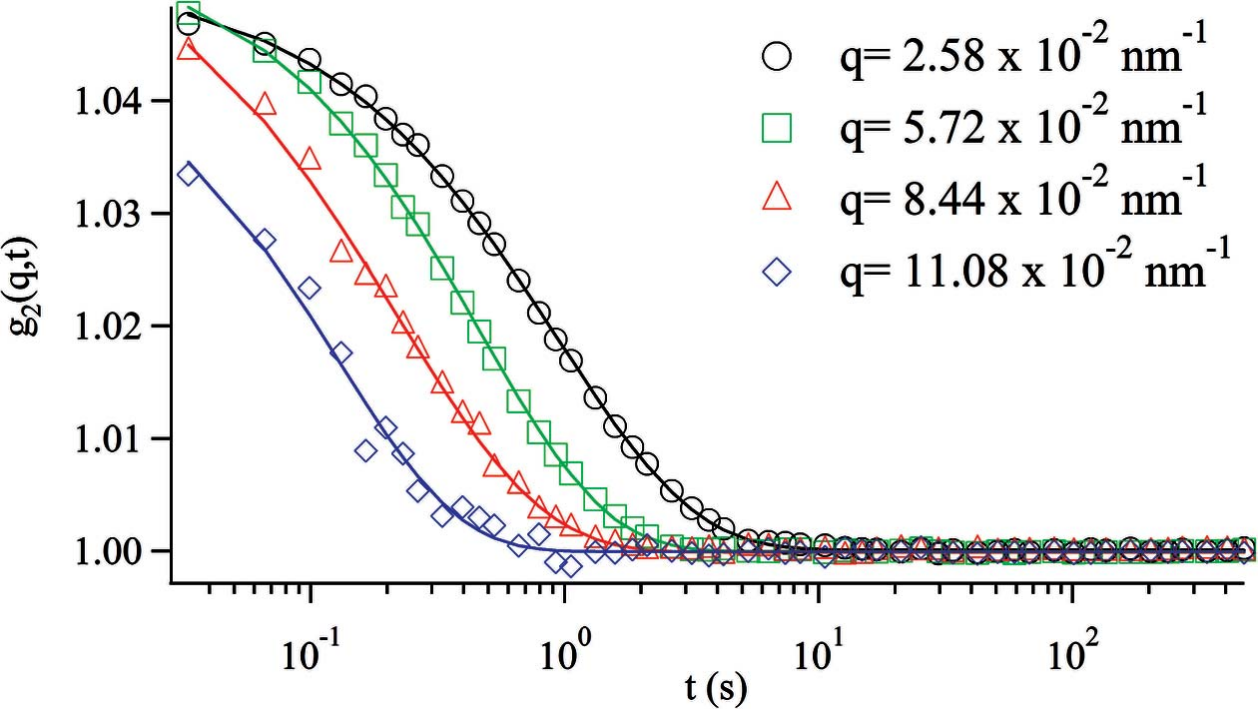
Normalized intensity time-autocorrelation function obtained from scattering of the PS grafted silica particles at 473.15 K. The solid lines are fitting curves by equation (6).

**Figure 7:**
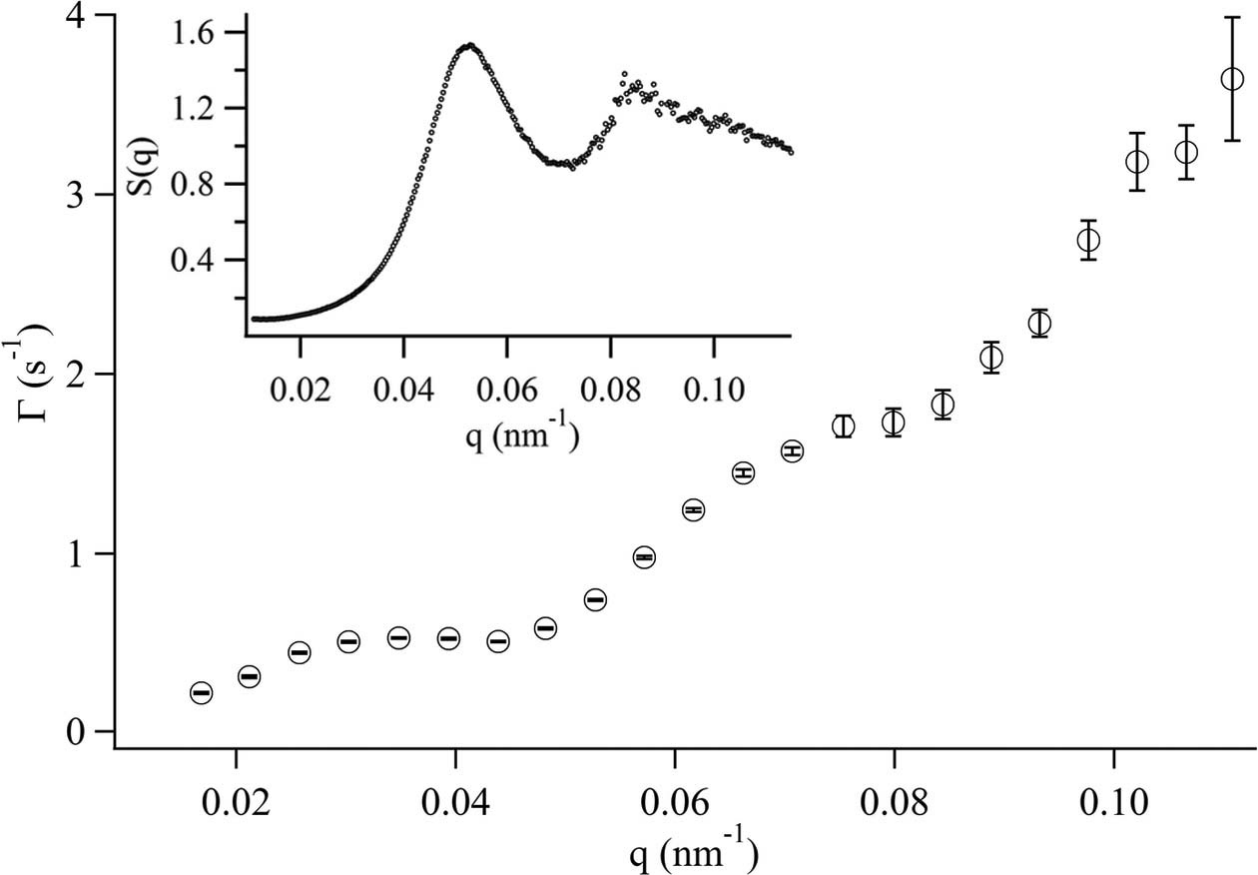
The *q*-dependence of relaxation rates Γ measured by XPCS for the PS grafted silica particles at 473.15 K. The static structure factor *S*(*q*) obtained by SAXS experiments is depicted in the inset.

Typical examples of measured *g*_2_(*q*, *t*) are shown in Fig. 6 ▶. All the measured data are fitted well by the stretched exponential form
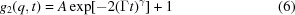
as shown by the solid lines. In this measurement the beam size was slightly reduced because of the small sample volume, thus the contrast, *A* ≃ 0.04, is higher than the data shown in the previous section. The relaxation rate Γ(*q*) is shown in Fig. 7 ▶, which has local minima around *q* = 5.0 × 10^−2^ nm^−1^ and *q* = 8.5 × 10^−2^ nm^−1^. From the static SAXS measurements the static structure factor *S*(*q*) was obtained, which has local maxima where *Γ*(*q*) has local minima as shown in the inset of Fig. 7 ▶. These results indicate that the XPCS measurements successfully observed so-called ‘de Gennes narrowing’, which is a generic feature of diffusing particles in concentrated suspensions demonstrating the slower decay of the most probable density fluctuations owing to caging by neighboring particles. Note that the DLS measurement cannot be used for samples as opaque as this one. The maximal measured *q* in this sample was *q* ≃ 0.111 nm^−1^, which is 3.5 times larger than that previously mentioned for the dilute particle system; the reason for this is the higher density of scatterers. These measurements demonstrate the performance of our XPCS system in a much higher *q* region, *i.e.* smaller length scale, than that measurable by DLS.

The measurable *q*-range and time scale should be varied with changing the hole size of the grid mask because the hole size affects the number of detected photons. At the maximum of measured *q*, the average number of pulses detected in a sampling time, 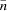, was equal to about 0.5. If the hole size was smaller than 45 µm in diameter, *e.g.* 15 µm in diameter, 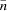 would be much less, and, therefore, the number of pulses detected in a sampling time is very often zero. Thus, most of the products carried out by using (1) are just zero and the measurable *q*-range or time scale would considerably decrease. According to Schatzel (1990 ▶), in the case of a single-exponential correlation function the fastest limit of measurable Γ is estimated by Γτ ≤ 0.1 where τ is the delay time. In our case we could measure up to Γ = 3.64 s^−1^ (at *q* = 0.111 nm^−1^) with a delay time of 33 ms (which is an exposure time of 30 ms and readout time of 3 ms), thus Γτ = 0.12, which is almost the limit estimated by Schatzel (1990 ▶). From these results, using a grid mask with 45 µm-diameter hole size seems to be a fairly good choice for accomplishing our purpose of measuring the fast dynamics at high *q*.

## 4. Conclusion

The performance of a fast pixel array detector with a grid mask resolution enhancer has been demonstrated for X-ray photon correlation spectroscopy (XPCS) measurements at beamline BL19LXU, SPring-8. The detector, a PILATUS 100K instrument, was mounted ∼3.3 m downstream of the sample, and each pixel was covered with a grid mask resolution enhancer consisting of a tantalum mask with holes ∼45 µm in diameter. For confirmation of the validity of our XPCS system in the small-*q* region, the dynamical behavior of silica particles dispersed in PPG4000 was measured, and the intensity time-autocorrelation function *g*_2_(*q*, *t*) was calculated from the intensity fluctuation. The *g*_2_(*q*, *t*) function was exponentially damped, and its behavior was expressed well by Brownian motion. The XPCS results were cross-checked using independent DLS measurements of the same sample, and the consistent results demonstrate the validity of our XPCS system. The XPCS system was also used to measure a concentrated particle system, PS grafted particles, that is opaque in visible light and cannot be measured by DLS. We obtained consistent data up to *q* ≃ 0.111 nm^−1^. Using our proposed method, XPCS measurements can be conducted by customizing the hole size of the grid mask resolution enhancer to suit the experimental conditions such as beam size, detector size and sample-to-detector distance. In the future the signal-to-noise ratio can be raised by narrowing the hole size of the grid mask when the higher flux of coherent X-rays can be utilized.
